# 8-Formylophiopogonanone *B* Antagonizes Paraquat-Induced Hepatotoxicity by Suppressing Oxidative Stress

**DOI:** 10.3389/fphar.2019.01283

**Published:** 2019-10-24

**Authors:** Jing-yu Qian, Ping Deng, Yi-dan Liang, Li Pang, Li-chuan Wu, Ling-ling Yang, Zhouv Zhou, Zheng-ping Yu

**Affiliations:** ^1^Department of Cell Biology, School of Life Sciences and School of Medicine, Guangxi University, Nanning, China; ^2^Department of Occupational Health, Third Military Medical University, Chongqing, China

**Keywords:** 8-formylophiopogonanone B, paraquat, hepatotoxicity, oxidative stress, apoptosis

## Abstract

Flavonoids are some of the most important natural products with a variety of physiological activities. 8-Formylophiopogonanone B (8-FOB) is a naturally existing homoisoflavonoid in Ophiopogon japonicus. Paraquat (PQ) has been widely used as a potent herbicide and has high toxicity in humans. The goal of the present study was to investigate whether 8-FOB could protect against PQ-induced hepatotoxicity in vitro and in vivo. We first tested the protective effects of 8-FOB on PQ-induced cytotoxicity in L02 cells by determining cell viability, intracellular oxidative stress levels, mitochondrial function, and apoptosis in vitro. To verify the protective effects of 8-FOB, we pretreated mice with 8-FOB and assessed liver function, hepatic oxidative stress, and histopathological changes after PQ administration. Our results revealed that 8-FOB could antagonize PQ-induced hepatotoxicity in vitro and in vivo. The antagonistic effects could be attributed to suppressing oxidative stress, preserving mitochondrial function, and inhibiting apoptosis. The present study is the first to document that 8-FOB, a homoisoflavonoid compound, is an effective antioxidant for antagonizing PQ-induced hepatotoxicity.

## Introduction

Paraquat (PQ, 4′-bipyridinium-1,1′-dimethyl-dichloride) is a non-selective herbicide that is widely used to control weeds and grasses. PQ is used in agricultural activities worldwide due to its low production costs, rapid action, and high toxicity, especially in developing countries (Suntres, 2002; Yadawa et al., 2019). Exposure to a single high dose of PQ by accidental exposure or intentional suicide usually causes damage to multiple organs and death. It has been well established that the accumulation of PQ is associated with lung, brain, kidney, liver, and heart damage (Sato et al., 1992; Hu et al., 2019). Clinical evidence suggests that the liver is one of the major target organs of PQ poisoning (Han et al., 2014; El-Boghdady et al., 2017). The liver is an essential organ in humans for metabolism and detoxification and is highly sensitive to oxidative damage. It is well known that drugs, environmental pollutants, and other xenobiotics can be transformed and metabolized to produce various types of free radicals in the liver. These excessive free radicals can cause oxidative damage to the liver (Awadalla, 2012; Zeinvand-Lorestani et al., 2018).

The molecular mechanisms underlying PQ-induced hepatotoxicity are not fully understood. It has been recognized that the toxicity of PQ is mainly mediated by its redox activity (Dragin et al., 2006). Previous studies have shown that PQ specifically inhibits the activity of mitochondrial complexes I and III, thereby disturbing the electron transfer chain and inhibiting the synthesis of NADPH (Cocheme and Murphy, 2008; Hou et al., 2019). The excess production of reactive oxidative species (ROS), such as nitric oxide and superoxide, has been observed in PQ intoxication, indicating that oxidative stress contributes to PQ-induced pathophysiological changes. Excessive free radicals produced by ROS could oxidize and destroy lipids in the cell membrane and hence lead to cell death (Zhang et al., 2019). Therefore, PQ-induced cellular oxidative stress is a prerequisite for its toxic effects. Immunosuppressants, blood dialysis, and some other treatment approaches are presently used to treat patients with PQ poisoning in clinical practice. However, there is still a lack of antagonistic drugs and specific treatment regimens. Existing clinical treatment for severe PQ intoxication can only alleviate symptoms and results in poor therapeutic effects with high mortality (Gawarammana and Buckley, 2011). Much effort has been made to develop new drugs to treat PQ toxicity in recent decades. Antioxidants have been proven to be effective in mitigating PQ-induced damage to organs when they are used in the early stages of intoxication. The screening and development of effective drugs for the treatment of PQ toxicity remains an important strategy for improving the clinical outcomes of PQ-poisoned patients.

Flavonoids, which are active ingredients in foods and are widely present in the human daily diet, are a class of compounds that have a flavonoid structure and are secondary metabolites of plants. Recently, flavonoids have received extensive attention due to their important biological activities, such as anti-tumor, antioxidation, anti-diabetic, antiviral, and immunomodulatory activities (D’Andrea, 2015; Jaroonwitchawan et al., 2017; Suntres, 2018). Many studies of nutritional intervention with flavonoids for chronic disease, such as diabetes, are further proof of the diversity of the biological activity of flavonoids. In a previous study, neohesperidin dihydrochalcone, a compound with a flavonoid structure, was proven to be effective against PQ-induced liver injury because of its antioxidative and anti-inflammatory effects (Shi et al., 2015). Therefore, flavonoids have high potential for development and utilization in antagonizing PQ toxicity. *Ophiopogon japonicus* is a traditional Chinese medicine that is widely distributed in China, Japan, and several countries in Southeast Asia. As an efficient economic crop in China, *O. japonicus* has long been used to make health teas to treat various diseases, such as pulmonary diseases and diabetes (Chen et al., 2016b; Zhao et al., 2017). 8-Formylophiopogonanone *B* (8-FOB) is a type of homoisoflavonoid that was recently isolated from the root tubers of *O. japonicus* (Zhou et al., 2013). To the best of our knowledge, the biological actions of 8-FOB remain to be elucidated. However, the efficacy and potency of its antioxidative effects are unclear. The determination of whether 8-FOB could antagonize PQ-induced hepatotoxicity by reducing ROS in the liver requires further testing.

In the present study, we used immortalized normal human hepatocytes (L02 cells) and male C57BL/6 mice for the first time to investigate whether 8-FOB could antagonize PQ-induced hepatotoxicity and to determine the potential protective mechanisms involved in 8-FOB activity. Our results indicated that 8-FOB reduces PQ-induced hepatotoxicity by suppressing oxidative stress.

## Materials and Methods

### Materials and Reagents

PQ dichloride was purchased from Sigma (St. Louis, MO, USA) and dissolved in distilled deionized water to produce a 1 M stock solution. The 1 M stock solution of PQ was diluted to the desired concentration with cell culture medium prior to use. 8-FOB (purity ≥ 98.0%) was a gift from the College of Pharmaceutical Sciences, Zhejiang University (Hangzhou, Zhejiang, China). 8-FOB dry powder was freshly dissolved in DMSO before use. The human hepatic cell line L02 was purchased from the Cell Resource Center at the Shanghai Institutes for the Biological Sciences, Chinese Academy of Sciences (Shanghai, China). All other chemicals and assay kits we have utilized here have been obtained as described below in detail.

### Cell Culture

The L02 cells were maintained in RPMI-1640 medium (Gibco, Thermo Fisher Scientific, USA) supplemented with 10% (*v*/*v*) fetal bovine serum (PAN, Germany) and antibiotics (100 μg/ml penicillin and 100 μg/ml streptomycin) (Gibco, Thermo Fisher Scientific, USA) in a humidified atmosphere with 5% CO_2_ at 37°C.

### Cell Viability Assay

Cell viability was evaluated by a colorimetric cell counting kit-8 assay (CCK-8, Dojindo, Japan). L02 cells were seeded in a 96-well microplate at a density of 8 × 10^3^ cells per well. To determine PQ toxicity, cells were incubated with different concentrations of PQ (250, 500, 750, and 1,000 μM), and cell viability was examined at 6, 12, 24, and 48 h post PQ treatment. To test the protective effect of 8-FOB against PQ toxicity, cells were treated with different concentrations of 8-FOB (2.5, 10, and 40 μM) for 6 h followed by treatment with 500 μM PQ for 24 h, and cell viability was assessed. All cell viability assays were detected by a microplate reader at 450 nm (Infinite M200 PRO, TECAN, Switzerland).

### Measurement of Total Intracellular and Mitochondrial ROS Generation

L02 cells were grown overnight in 96-well microplates and pretreated with 8-FOB for 6 h. The production of mitochondrial ROS (mtROS) and total intracellular ROS was measured by the MitoSOX™ Red Mitochondrial Superoxide Indicator (Molecular Probes, Thermo Fisher Scientific, USA) (Chen et al., 2016a) and CM-H2DCFDA (Invitrogen, Thermo Fisher Scientific, USA) (Maddirala et al., 2015) 3 h after 500 μM PQ exposure, respectively. Cells were loaded with MitoSOX™ Red Mitochondrial Superoxide Indicator or CM-H2DCFDA, incubated at 37°C for 30 min and washed twice in phosphate-buffered saline (PBS) (Beyotime Institute of Biotechnology, Shanghai, China), and the fluorescence values were determined according to the manufacturer’s instructions.

### Determination of the Mitochondrial Membrane Potential (Δψm)

The mitochondrial membrane potential (ΔΨm) was assessed using the JC-1 Assay Kit (Beyotime Institute of Biotechnology, Shanghai, China) according to the manufacturer’s instructions (Zhang et al., 2014a). L02 cells were cultured in 96-well black microplates overnight. The cells were treated with 500 μM PQ for 24 h. A total of 100 μl JC-1 test solution (1 μg/ml) was added to each well and incubated for 20 min at 37°C in the dark. At the end of the incubation, the cells were washed twice with Hank’s Balanced Salt Solution (HBSS). The intensity of the red and green fluorescence was determined using a microplate reader at 560 nm excitation/590 nm emission and 488 nm excitation/525 nm emission, respectively.

### Measurement of ATP Levels

Intracellular ATP levels were measured 24 h after different treatments. The ATP levels were quantified using an ATP Determination Kit (Molecular Probes, Thermo Fisher Scientific, USA) (Yang et al., 2002). All solutions used for the assay were freshly prepared according to the manufacturer’s instructions. The reaction solution was added to a 96-well microplate (100 μl per well), incubated in 5% CO_2_ at 37°C for 15 min, and then the luminescence intensity was immediately detected.

### Determination of Lipid Peroxidation

Malondialdehyde (MDA) is a thiobarbituric acid (TBA) reactive (TBAR) material. The extent of cellular lipid peroxidation was determined by measuring the concentration of the TBA–MDA complex (Mao et al., 2012). Briefly, cells exposed to different treatments were harvested with trypsin and lysed on ice for 30 min with radioimmunoprecipitation assay (RIPA) lysis buffer. After centrifugation at 20,000 *g* for 30 min at 4°C, the supernatant was collected for the detection of the total protein concentration, and the MDA level was measured by using an MDA Assay Kit (Nanjing Jiancheng Bioengineering Institute, China) according to the manufacturer’s instructions.

### Caspase-3 Activity Assay

Caspase-3 activity was determined to assess apoptosis in L02 cells using the GreenNuc™ Caspase-3 Assay Kit (Beyotime, Shanghai, China). L02 cells were cultured in 96-well black microplates. After different treatments, 100 μl Ac-DEVD-CHO (10 μM; a caspase-3/7 inhibitor) and 100 μl GreenNuc™ Caspase-3 Substrate (5 μM) were added and incubated at room temperature for 30 min. The fluorescence was determined at 485 nm excitation and 515 nm emission using a microplate reader (Infinite M200 PRO, TECAN, Switzerland).

### Flow Cytometric Analysis

L02 cells (1 × 10^5^ cells per well) were seeded in a six-well microplate. After different treatments, the cells were harvested and washed twice with pre-cooled Dulbecco’s PBS (D-PBS), resuspended in 200 μl of binding buffer containing 3 μl propidium iodide (PI) and 3 μl annexin V–fluorescein isothiocyanate (FITC) and incubated for 15 min in the dark. All of the samples were analyzed immediately by a flow cytometer (BD Accuri C6, USA) (Li et al., 2010).

### Western Blot Analysis

Cultured cells were washed with ice-cold PBS and lysed in a buffer containing RIPA and 1% protease inhibitor cocktail (Roche, Switzerland). The cell lysates were centrifuged at 20,000 *g* for 30 min at 4°C, and the supernatants were collected; the protein concentrations were determined by the BCA Protein Assay Kit (Beyotime, Shanghai, China). Cell lysates containing 50 μg protein were loaded and resolved by sodium dodecyl sulfate–polyacrylamide gel electrophoresis (SDS-PAGE) and transferred to polyvinylidene difluoride (PVDF) membranes according to standard procedures. The membranes were washed in Tris-buffered saline (T-TBS) and blocked for 2 h with PBS containing 5% nonfat milk, and the membranes were then incubated with primary antibodies against caspase-3 (1:1,000, Invitrogen, Thermo Fisher Scientific, USA) (Tian et al., 2015) and β-actin (1:1,000; Sigma, USA) overnight at 4°C. The membranes were washed three times with T-TBS for 10 min each time prior to incubation with the secondary antibody. The signals were detected with Western chemiluminescent horseradish peroxidase (HRP) substrate in accordance with the manufacturer’s instructions. After the film was scanned with a ChemiDoc™ XRS+ imaging densitometer (Bio-Rad, USA), quantitative analysis was performed using Image Lab™ software.

### Animal Experiments

Male C57BL/6 mice (20–24 g) aged 8 weeks were purchased from the animal center of the Army Medical University (Chongqing, China). All animals were allowed 1 week for acclimatization before being used in experiments. The animals were housed in a room with a 12-h light/dark cycle and standard laboratory food and fresh water *ad libitum* during the entire experiment. The animal experiments were approved by the Third Military Medical University Animal Care and Use Committee. PQ was dissolved in saline for intraperitoneal injection. 8-FOB dry powder was ultrasonically mixed into a homogeneous suspension in a 0.5% carboxymethylcellulose sodium (CMC-Na) solution for intragastric administration. The mice were randomly divided into five groups (*n* = 5): control group, solvent (0.5% CMC-Na) group, 8-FOB group, PQ group, and 8-FOB + PQ group. The mice in the control group were fed by gavage an equal volume of normal saline. The mice in the solvent group were fed by gavage an equal volume of 0.5% CMC-Na (Wu et al., 2019), while the mice in the 8-FOB group were fed by gavage 20 mg/kg/day 8-FOB. In the 8-FOB + PQ group, the mice were pretreated by gavage with 20 mg/kg/day 8-FOB for three consecutive days and then injected with a single dose of 30 mg/kg PQ intraperitoneally. All mice in the different groups were sacrificed 24 h after treatment. Blood and liver samples were collected for biochemical and histological analyses.

### Serum Biochemistry Analysis

The blood samples were kept at room temperature for 2 h. The serum was then collected after centrifugation at 3,000 *g* for 10 min and stored at −80°C until the liver function tests were performed. The activity of alanine transaminase (ALT) and aspartate transaminase (AST) in serum was measured with commercial kits according to the manufacturer’s instructions.

### Liver Histopathology Study

Histological examination of liver tissue was used to assess the hepatic damage caused by PQ and the protective effect of 8-FOB. Liver specimens were fixed in 4% paraformaldehyde, embedded in paraffin, cut into 4-μm-thick sections, and stained with hematoxylin and eosin (H&E). Morphological observation was conducted using a microscope (Eclipse Ci, Nikon, Japan). Representative images were obtained. Histopathological analysis of hepatic lesions was performed quantitatively, and injury score was graded according to Suzike’s criteria (Ge et al., 2017).

### Assays of Oxidative Stress Parameters

The collected liver tissue was washed with ice-cold saline solution, immediately frozen in liquid nitrogen, and stored at −80°C for biochemical analysis. The kits for the determination of superoxide dismutase (SOD), glutathione (GSH), catalase (CAT), and MDA activity were obtained from the Nanjing Jiancheng Institute of Biotechnology (Nanjing, China). The SOD kit detected the activity of total SOD (T-SOD) according to the xanthine oxidase method (Mao et al., 2010). The xanthine–xanthine oxidase system produces superoxide ions that react with 2-(4-iodophenyl)-3-(4-nitrophenol-5-phenyltetrazolium chloride) to form a red formazan dye, and the absorbance at 550 nm was determined. Hydrogenase (CAT) decomposes H_2_O_2_ into H_2_O and O_2_. The CAT detection kit rapidly terminates the decomposition of H_2_O_2_ by the addition of ammonium molybdate. The remaining H_2_O_2_ reacts with the ammonium molybdate to form a yellow complex that can be measured at 405 nm. The amount of CAT can be calculated from the absorbance value (Zhang et al., 2014b). Glutathione (GSH) is the major non-protein sulfhydryl compound in tissues. It reacts with dithiodinitrobenzoic acid (DTNB) to form thionitrobenzoic acid. According to the principle underlying the assay, the amount of GSH can be calculated by quantitatively measuring the absorbance at 405 nm of thionitrobenzoic acid (Beutler et al., 1963). The concentration of MDA in liver tissue was used to assess the lipid peroxidation levels. MDA reacts with TBA at a high temperature in an acidic environment to form a red MDA–TBA complex with a maximum absorbance at 532 nm, and the concentration of MDA is calculated from the measured absorbance values (Liu et al., 2013).

### Statistical Analysis

The statistical analysis was carried out by using GraphPad Prism 5.0, and the data are presented as the mean ± standard error. One-way ANOVA followed by the Tukey multiple comparison test was employed to test the significance.

## Results

### Dose- and Time-Dependent Cytotoxicity of PQ in L02 Cells

**Figure 1:**
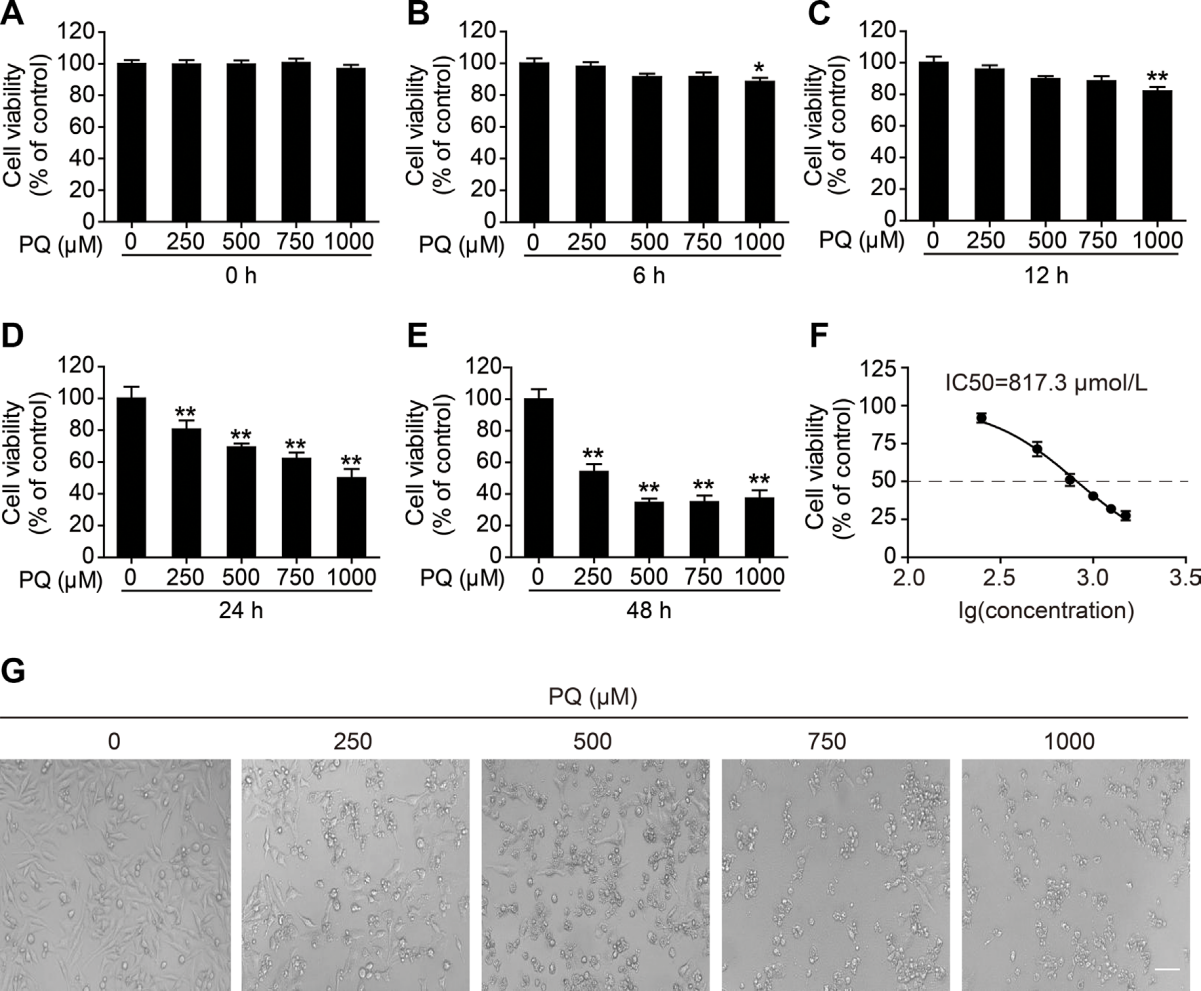
Dose- and time-dependent cytotoxicity of PQ in L02 cells. Cell viability was measured by a cell counting kit (CCK)-8 assay **(A)** 0, **(B)** 6, **(C)** 12, **(D)** 24, and **(E)** 48 h after treatment with the indicated concentrations of PQ. **(F)** The mean inhibitory concentration (IC50) fit curve was calculated according to PQ exposure for 24 h and the measurement of the inhibition of cell viability. The values are presented as the means ± SEM (*n* = 8). *p < 0.05, **p < 0.01 vs control group. **(G)** Morphological observation of PQ-induced cell damage detected by light microscopy. Scale bar: 80 μm.

To determine the cytotoxicity of PQ in L02 cells, the cell viability at different time points after PQ treatment was examined. A dose- and time-dependent decrease in cell viability was observed in L02 cells upon exposure to PQ (**Figures 1A–E**). Treatment with 1,000 μM PQ significantly reduced cell viability at 6 h post exposure, while PQ concentrations lower than 1,000 μM did not result in a reduction in cell viability at 6 h post exposure (**Figure 1B**). Cell viability was markedly decreased at 24 h post exposure at all tested concentrations (**Figure 1D**) and was further decreased at 48 h post exposure (**Figure 1E**). The concentration of 500 μM used as the toxic exposure level in the following experiments induced a significant reduction in cell viability to 71.8%, as determined at 24 h post induction of L02 toxicity and shown in **Figure 1D**. The 50% inhibitory concentration (IC50) of PQ in L02 cells after 24 h of exposure was calculated by GraphPad Prism 5.0 to be 817.3 μM (**Figure 1F**). Furthermore, we observed dose-dependent morphological damage after exposure to different concentrations of PQ by using a microscope (Eclipse Ci, Nikon, Japan) to examine L02 cells (**Figure 1G**).

### 8-FOB Suppresses PQ-Induced Cytotoxicity in L02 Cells

**Figure 2:**
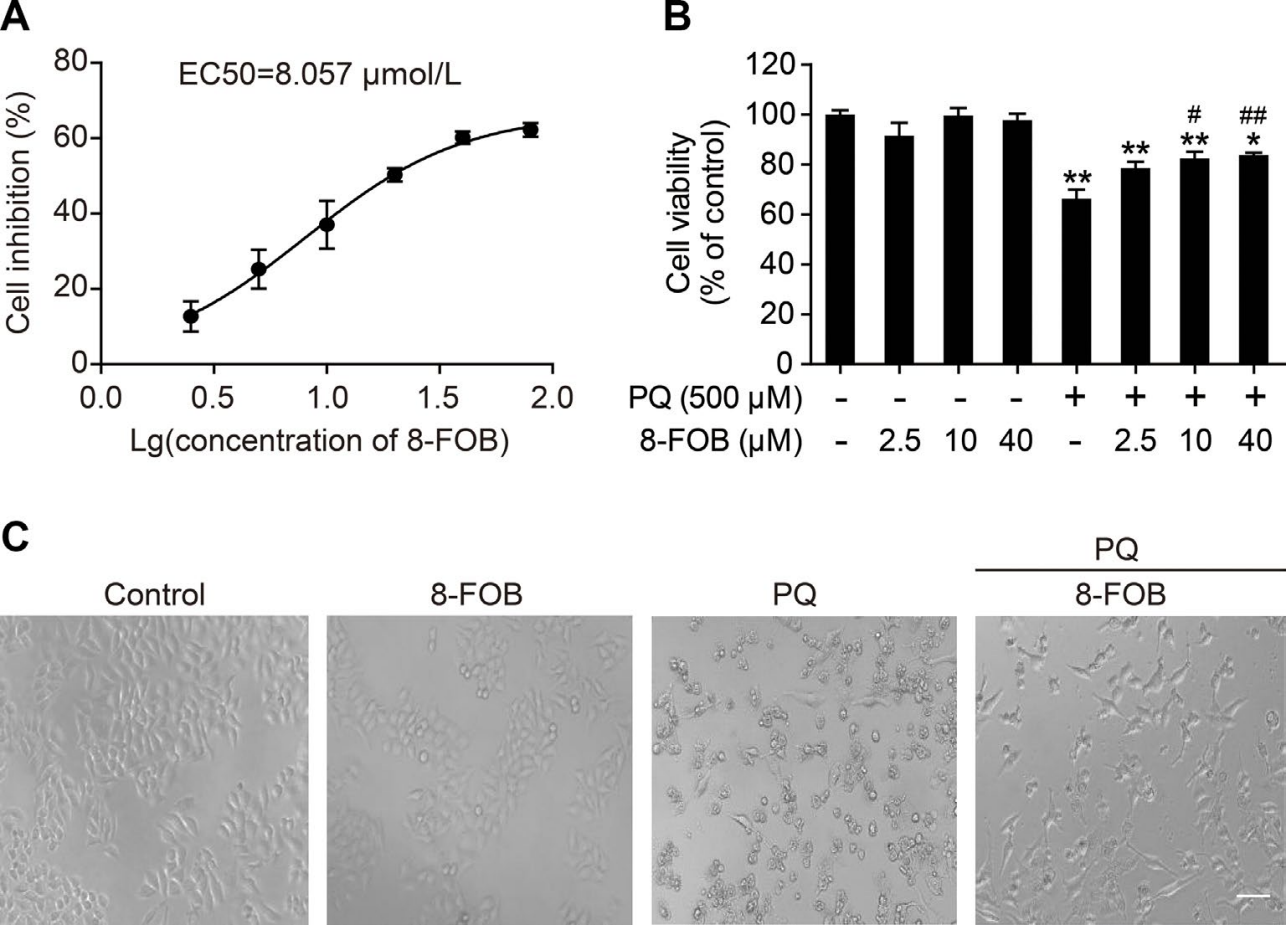
8-Formylophiopogonanone *B* (8-FOB) suppresses paraquat (PQ)-induced cytotoxicity in L02 cells. **(A)** Fitted curve showing the inhibition of cytotoxicity by 8-FOB. The EC50 value of 8-FOB for the protection against PQ toxicity in L02 cells was 8.057 μM. **(B)** The effect of pretreatment with different 8-FOB concentrations on cell viability in the presence of 500 μM PQ. The values are presented as the means ± SEM (*n* = 6). *p < 0.05, **p < 0.01 vs control group; ^#^ *p* < 0.05, ^##^ *p* < 0.01 vs the PQ group. **(C)** Representative images showing the effects of 8-FOB pretreatment on cell damage induced by 500 μM PQ. Scale bar: 80 μm.

To investigate the protective effect of 8-FOB on PQ-induced toxicity in L02 cells, we pretreated cells with 2.5, 10, and 40 μM 8-FOB for 6 h and then incubated the cells with 500 μM PQ for 24 h to determine cell viability. The effective concentration that produced a 50% response (EC50) for 8-FOB in L02 cells was calculated to be 8.057 μM based on the results of the cell viability assay (**Figure 2A**). 8-FOB treatment alone did not affect cell viability at the three indicated concentrations (**Figure 2B**), suggesting that there is no cytotoxic effect of 8-FOB treatment at the high concentration of 40 μM (approximately five times higher than the EC50). PQ treatment at 500 μM for 24 h induced the decline of cell viability to 73.4%. Although 8-FOB pretreatment failed to restore the cell viability to the control level, pretreatment with 10 and 40 μM 8-FOB significantly elevated cell viability (**Figure 2B**), indicating that 8-FOB reduced PQ-induced cytotoxicity. In addition, we found that pretreatment with 40 μM 8-FOB clearly provided protection against morphological damage induced by 500 μM PQ according to the morphological observations (**Figure 2C**).

### 8-FOB Antagonizes PQ-Induced Oxidative Stress in L02 Cells

**Figure 3:**
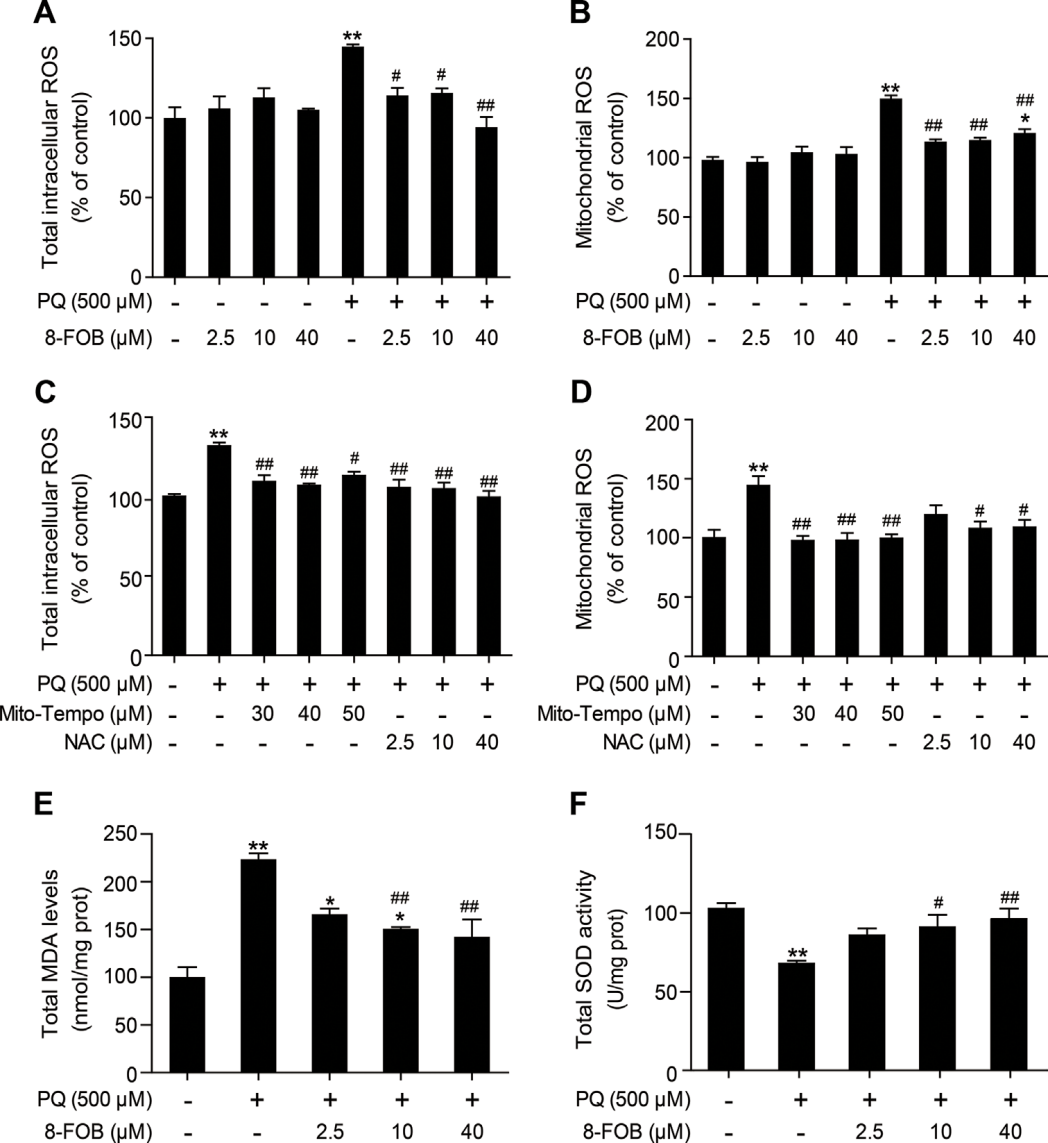
8-Formylophiopogonanone *B* (8-FOB) reduces paraquat (PQ)-induced oxidative stress in L02 cells. Total intracellular reactive oxygen species (ROS) **(A, C)** and mitochondrial ROS **(B, D)** were detected 3 h after PQ treatment in L02 cells. The total malondialdehyde (MDA) level **(E)** and superoxide dismutase (SOD) activity **(F)** were measured 24 h after PQ treatment. The values are presented as the means ± SEM (*n* = 6). *p < 0.05, **p < 0.01 vs the control group; ^#^ *p* < 0.05, ^##^ *p* < 0.01 vs the PQ group.

PQ is a well-known potent inducer of ROS. ROS production and cellular oxidative stress have been proven to be the early events that initiate pathophysiological changes. In an attempt to assess whether 8-FOB could protect L02 cells against PQ toxicity, we measured total intracellular ROS and mtROS levels, which indicated that oxidative stress is present as early as 3 h after PQ treatment. Our results demonstrated that treatment with 8-FOB alone did not alter the total intracellular ROS and mtROS levels. Pretreatment with 2.5, 10, and 40 μM 8-FOB for 6 h markedly inhibited both total intracellular ROS and mtROS production, as determined at 3 h post PQ exposure (**Figures 3A, B**). Furthermore, we used Mito-TEMPO, a mitochondrion-targeted antioxidant with superoxide and alkyl radical scavenging properties (Du et al., 2017), and *N*-acetyl--cysteine (NAC), a scavenger of total intracellular ROS (Ahmad et al., 2013), to verify the existence of the PQ-induced production of intracellular ROS and mtROS. Treatments with these two scavengers significantly reduced the total intracellular ROS and mtROS levels (**Figures 3C, D**). MDA, a free radical-mediated metabolite of lipid peroxidation, is widely used as a marker of oxidative stress. PQ treatment resulted in a significant increase in MDA levels compared with those in the control. However, 8-FOB pretreatment markedly reduced MDA levels compared with those resulting from PQ treatment alone (**Figure 3E**). SOD activity is a well-defined biomarker of cellular oxidative stress. We found that PQ treatment caused a marked reduction in total intracellular SOD activity compared with that in the control, and the suppressive effect of PQ treatment on SOD activity could be significantly counteracted by 8-FOB pretreatment (**Figure 3F**). Overall, these data suggest that 8-FOB reduces PQ-induced oxidative stress in L02 cells.

### 8-FOB Alleviates PQ-Induced Mitochondrial Dysfunction in L02 Cells

**Figure 4:**
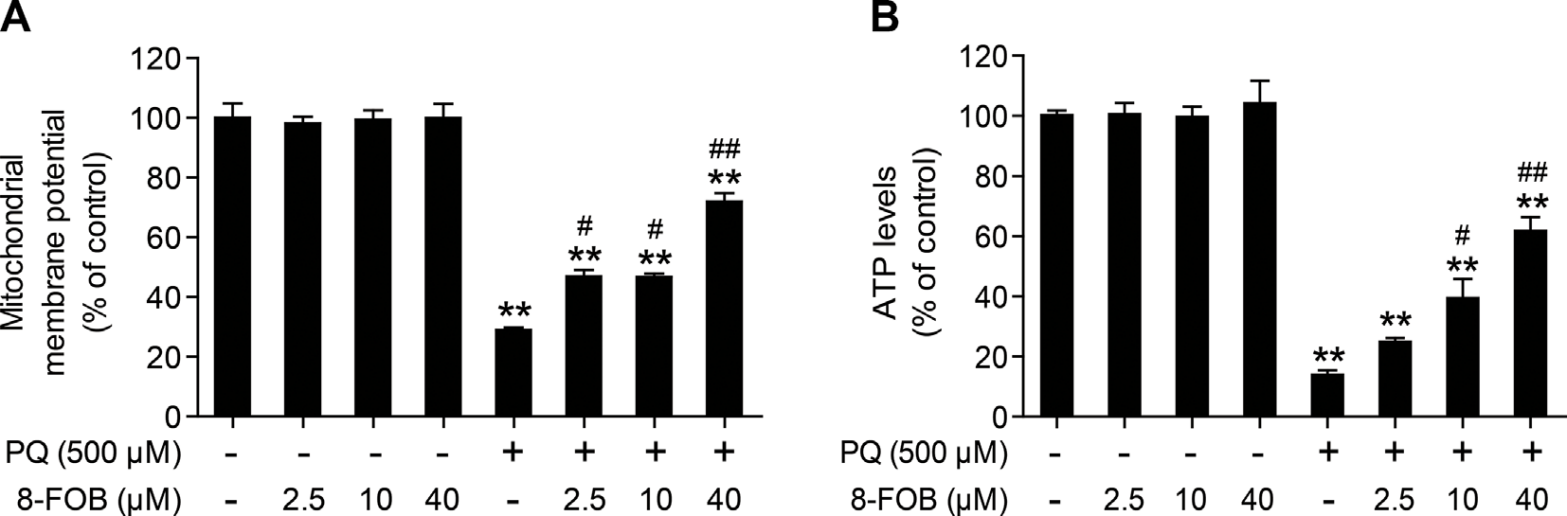
8-Formylophiopogonanone *B* (8-FOB) alleviates paraquat (PQ)-induced mitochondrial dysfunction in L02 cells. **(A)** The mitochondrial membrane potential (ΔΨm) of L02 cells was measured 24 h after PQ treatment. **(B)** Measurement of ATP levels after 24 h of PQ treatment. The values are presented as the means ± SEM (*n* = 6). **p < 0.01 vs the control group; ^#^ *p* < 0.05, ^##^ *p* < 0.01 vs the PQ group.

To maintain normal mitochondrial function, it is essential to maintain intracellular ROS at the physiological level. It is generally accepted that the elevation of intracellular ROS and mtROS production during cellular oxidative stress could impair mitochondrial function. Since 8-FOB could remarkably reduce ROS production and cellular oxidative stress, we further explored whether 8-FOB could protect against PQ-induced mitochondrial dysfunction, as indicated by the alteration of the mitochondrial membrane potential and the decline in cellular ATP levels. We measured the effect of 8-FOB on the mitochondrial membrane potential (ΔΨm) and ATP levels in the presence and absence of PQ. As shown in **Figure 4A**, 8-FOB treatment did not affect the mitochondrial membrane potential (ΔΨm) at the three tested concentrations, while PQ treatment significantly decreased the mitochondrial membrane potential (ΔΨm). 8-FOB pretreatment at the three tested concentrations markedly reduced the decrease in the mitochondrial membrane potential (ΔΨm) compared with PQ treatment alone. Intracellular ATP concentration is a sensitive functional indicator for mitochondria. We found that 8-FOB treatment alone had no effect on intracellular ATP levels, while PQ exposure markedly decreased intracellular ATP levels. 8-FOB pretreatment at the three tested concentrations markedly reduced the PQ-induced decreases in intracellular ATP concentrations, as shown in **Figure 4B**. These results suggested that 8-FOB could markedly attenuate PQ-induced mitochondrial dysfunction though suppressing intracellular oxidative stress.

### 8-FOB Mitigates PQ-Induced Apoptosis in L02 Cells

**Figure 5:**
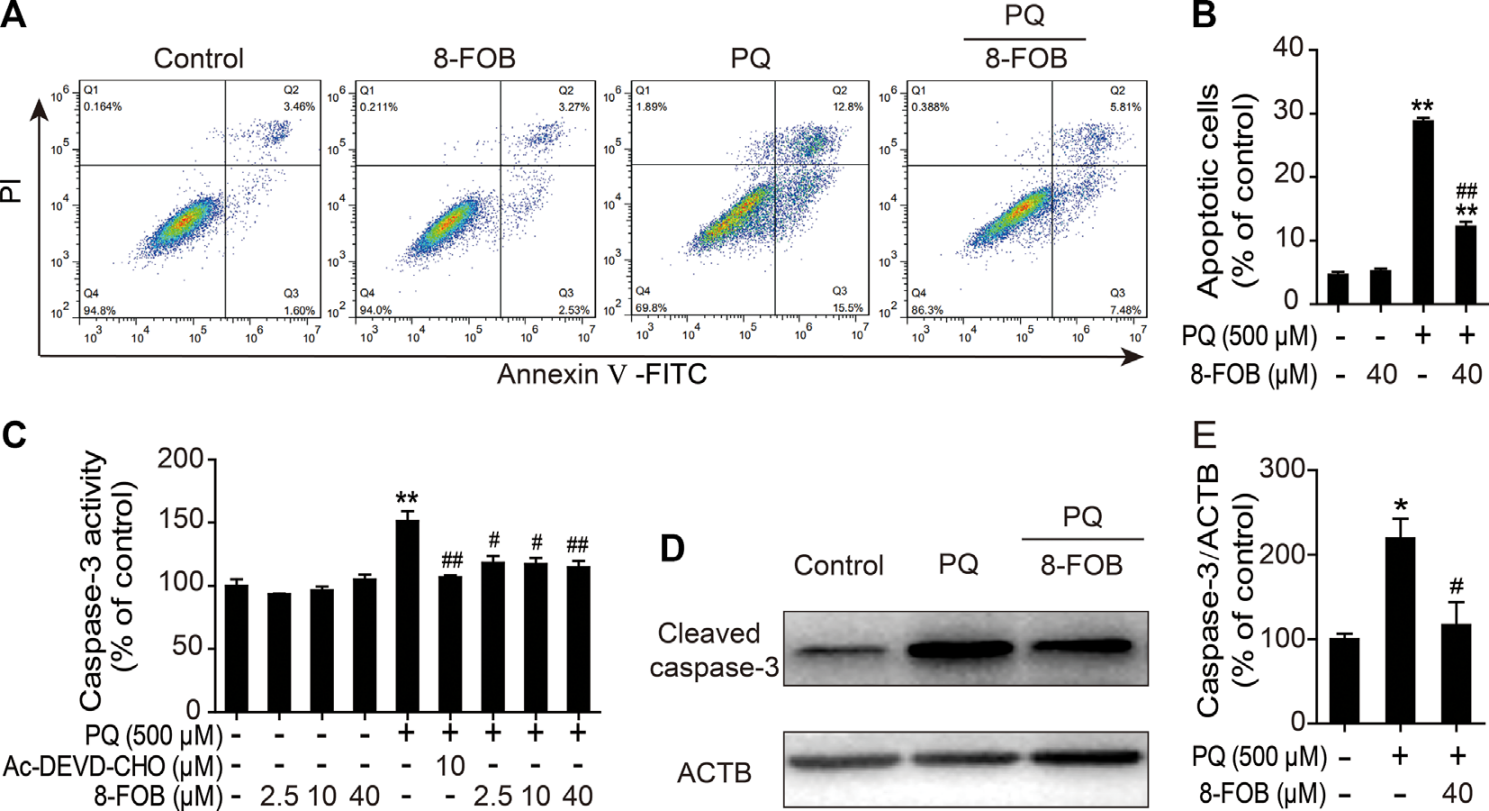
8-Formylophiopogonanone *B* (8-FOB) mitigates paraquat (PQ)-induced apoptosis in L02 cells. **(A)** Representative images showing the effects of 8-FOB inhibition on PQ-induced apoptosis determined by flow cytometry. The annexin V–fluorescein isothiocyanate (FITC)−/propidium iodide (PI)− population consists of normal healthy cells (Q4); annexin V–FITC+/PI− cells indicate early apoptosis (Q3); annexin V–FITC+/PI+ cells indicate late apoptosis or necrosis (Q2); annexin V–FITC−/PI+ cells are necrotic cells (Q1). **(B)** Quantification of apoptosis ratios. **(C)** Detection of caspase-3 activity after 24 h of PQ (500 μM) treatment. Ac-DEVD-CHO: a specific caspase-3 inhibitor. **(D)** Detection of caspase-3 expression levels after 24 h of PQ (500 μM) treatment. **(E)** Quantification of cleaved caspase-3 levels relative to those of β-actin. The values are presented as the means ± SEM (*n* = 6). *p < 0.05, **p < 0.01 vs the control group; ^#^ *p* < 0.05, ^##^ *p* < 0.01 vs the PQ group.

It has been reported that apoptosis plays an important role in PQ-induced damage in the lung, liver, kidney, and other organs and that cellular oxidative stress is one of the major potent inducers of apoptosis. Therefore, we investigated whether the protective effect of 8-FOB on PQ cytotoxicity occurs by inhibiting apoptosis. Apoptosis was measured by flow cytometry after staining cells with annexin V/PI probes. We found that the proportion of apoptotic cells was markedly increased in PQ-treated L02 cells. Pretreatment with 40 μM 8-FOB significantly reduced the PQ-induced increase in the apoptotic rate (**Figures 5A, B**). Active cleaved caspase-3 plays a pivotal role in the induction of apoptosis. Treatment with 8-FOB at the three indicated concentrations did not affect caspase-3 activity, while PQ treatment significantly increased caspase-3 activity. Pretreatment with 8-FOB at the three indicated concentrations markedly suppressed the elevation of PQ-induced caspase-3 activity. The suppressive effect of 8-FOB on caspase-3 activity in the presence of PQ was comparable to that of a specific inhibitor of caspase-3 activity, Ac-DEVD-CHO (**Figure 5C**). In the Western blotting analysis, we found that the protein expression of active cleaved caspase-3 was obviously upregulated in PQ-exposed L02 cells. Pretreatment with 8-FOB inhibited the PQ-induced upregulation of active cleaved caspase-3 (**Figures 5D, E**). Overall, these data suggested that 8-FOB could suppress PQ-induced apoptosis.

### 8-FOB Reduces PQ-Induced Liver Injury in Mice

**Figure 6:**
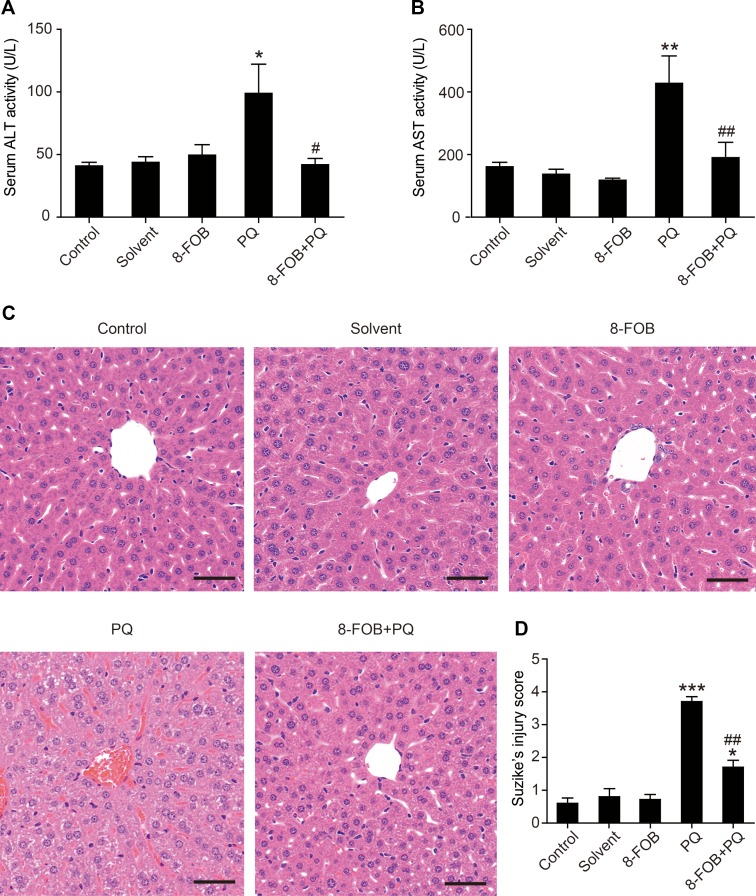
8-Formylophiopogonanone *B* (8-FOB) reduces paraquat (PQ)-induced liver injury in mice. Serum alanine transaminase (ALT) **(A)** and aspartate transaminase (AST) **(B)** activities determined at 24 h after PQ injection. The values are presented as the means ± SEM (*n* = 5). *p < 0.05, **p < 0.01 vs the control group; ^#^ *p* < 0.05, ^##^ *p* < 0.01 vs the PQ group. **(C)** Representative images of the morphological observations of histopathological changes. Scale bar: 50 μm. **(D)** Suzike’s score of hepatic lesions analyzed quantitatively. The values are presented as the means ± SEM (*n* = 10). *p < 0.05, ***p < 0.001 vs the control group; ^##^ *p* < 0.01 vs the PQ group.

To test whether 8-FOB could provide *in vivo* protection against PQ hepatotoxicity, we next conducted an animal study to examine the efficacy of 8-FOB pretreatment on PQ-induced liver injury in mice. A single dose of 30 mg/kg PQ administered by intraperitoneal injection resulted in obvious liver injury, as determined at 24 h post PQ injection and indicated by the marked elevation of serum aspartate aminotransferase (AST) and alanine aminotransferase (ALT), both of which are serum markers of liver function. When mice were given 20 mg/kg/day 8-FOB pretreatment by gavage for three consecutive days, PQ-induced elevations in AST and ALT were significantly suppressed at 24 h post PQ injection (**Figures 6A, B**). Furthermore, a single dose of 30 mg/kg PQ administrated by intraperitoneal injection resulted in necrosis, cell shrinkage, the congestion of portal areas, and sinusoidal hemorrhage in the liver. Pretreatment with 20 mg/kg/day 8-FOB for three consecutive days by gavage markedly attenuated PQ-induced hepatic histopathological lesions indicated by the significantly lower Suzike injury score in the 8-FOB pretreatment group (**Figures 6C, D**). The results from this *in vivo* study demonstrated that 8-FOB effectively mitigated PQ-induced liver injury functionally and morphologically.

### 8-FOB Attenuates PQ-Induced Oxidative Stress in the Liver of Mice

**Figure 7:**
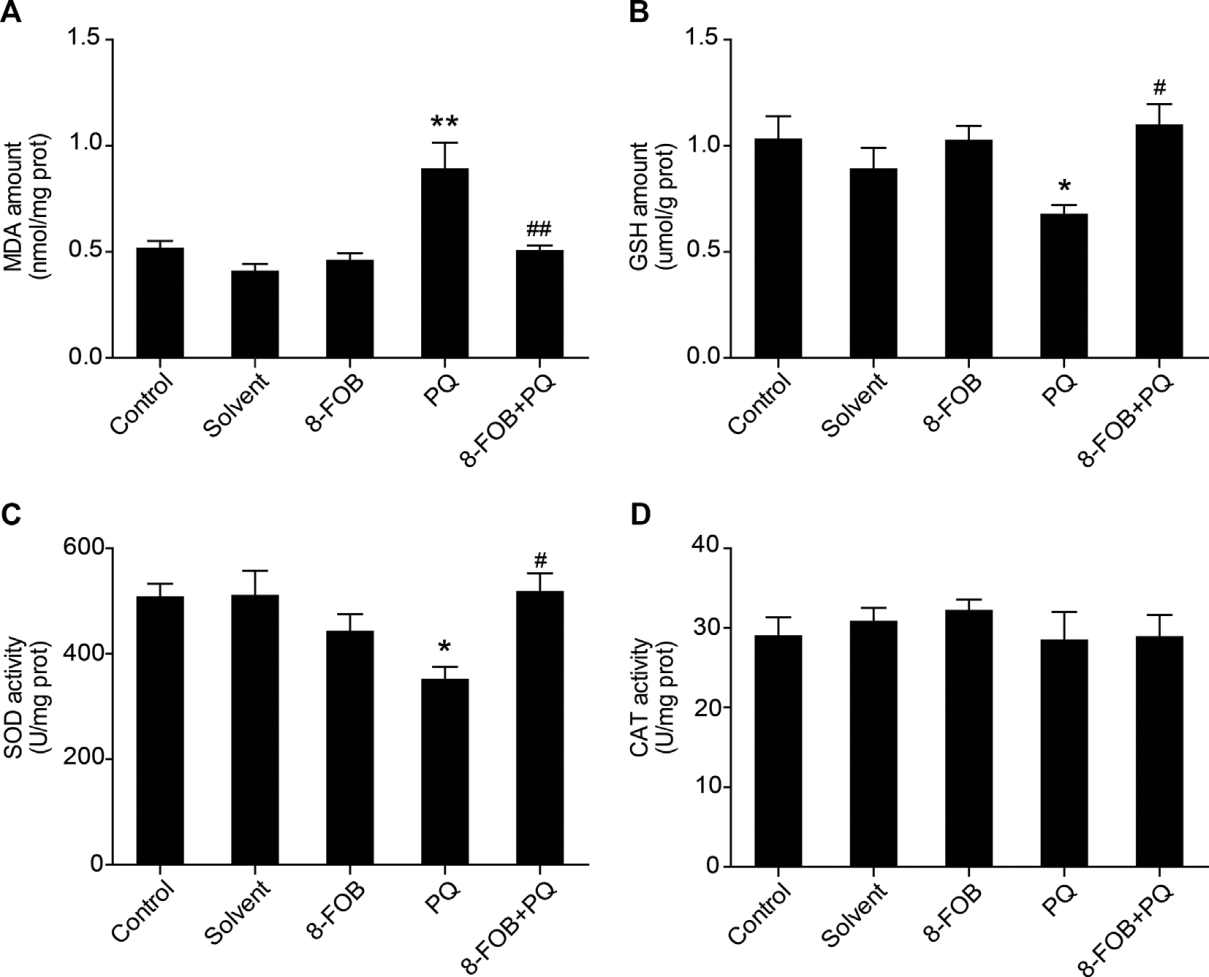
8-Formylophiopogonanone *B* (8-FOB) attenuates paraquat (PQ)-induced hepatic oxidative stress in mice. Changes in the malondialdehyde (MDA) amount **(A)**, glutathione (GSH) content **(B)**, superoxide dismutase (SOD) activity **(C)**, and catalase (CAT) activity **(D)** in the liver. The values are presented as the means ± SEM (*n* = 5). *p < 0.05, **p < 0.01 vs the control group; ^#^ *p* < 0.05, ^##^ *p* < 0.01 vs the PQ group.

To examine whether 8-FOB could inhibit hepatic oxidative stress *in vivo*, we pretreated mice with 8-FOB 20 mg/kg/day for three consecutive days and then administered PQ 30 mg/kg intraperitoneally. We found that treatment with 8-FOB alone did not induce obvious changes in hepatic oxidative stress, while PQ administration resulted in remarkably increased levels of hepatic oxidative stress, as indicated by the significant elevation in MDA levels and the marked decreases in GSH and SOD levels. Pretreatment with 8-FOB significantly suppressed the PQ-induced elevation in MDA levels and attenuated the decreases in GSH and SOD levels (**Figures 7A–C**). There was no significant alteration in CAT activity in the different treatment groups, indicating that CAT activity is not a sensitive indicator of oxidative stress during PQ intoxication (**Figure 7D**). These results suggested that 8-FOB effectively inhibited PQ-induced hepatic oxidative stress.

## Discussion

The present study, which is based on antagonistic experiments of cytotoxicity *in vitro* and protective investigations *in vivo*, is the first to document that (i) 8-FOB, a homoisoflavonoid compound, is an effective antioxidant for reducing PQ-induced hepatotoxicity; (ii) the inhibition of hepatic oxidative stress, which is characterized by the suppression of MDA production and intracellular ROS and mtROS generation and the reduction of GSH and SOD levels, is a key factor in 8-FOB-induced protection against PQ-induced hepatotoxicity; and (iii) 8-FOB, which has an EC50 of 8.057 μM *in vitro*, does not show cytotoxic effects even when administered at a much higher dose *in vitro* and *in vivo*. This demonstrates the potential of 8-FOB for future clinical use.

PQ is a common herbicide that causes high mortality after poisoning due to a lack of effective therapeutic drugs and regimens. Therefore, it is necessary to further screen and develop drugs that have the potential to antagonize PQ toxicity. Although the specific mechanism involved in PQ-induced toxicity is not fully understood, previous studies have shown that an important contributor to PQ poisoning is the excessive induction of ROS, which leads to a loss of balance in the redox system, thereby activating the intracellular signaling cascade and leading to oxidative stress, the impairment of mitochondrial function, and the induction of apoptosis (Bus et al., 1976; Jang et al., 2015).

In the past decade, flavonoids have received increasing attention due to their strong health-promoting properties. As signal molecules with diverse biological functions in plants, flavonoid compounds have recently been widely used in disease treatment and chemoprevention studies (Panche et al., 2016; Xu et al., 2017). In view of the important role of cellular oxidative stress in the mechanisms underlying PQ intoxication, flavonoids and their active derivatives, the homoisoflavonoid compounds, are reasonably considered to be potential targets for developing drugs for PQ intoxication due to their potent antioxidative effects. Previous phytochemical investigations have revealed that *O. japonicus* is rich in homoisoflavonoids and 8-FOB is the major constituent of the total homoisoflavonoid content. However, there is a lack of information on the biological and pharmacological activities of 8-FOB. The information that has been reported regarding its chemical nature is mostly irrelevant to its biological functions.

The present study aimed to investigate whether 8-FOB could antagonize PQ-induced hepatotoxicity and to reveal how its protective effects are exerted. First, we found that PQ-induced cytotoxicity *in vitro* is characterized by the production of intracellular ROS and mtROS and lipid peroxidation, indicating the existence of oxidative stress in the early stage of intoxication. Several previously reported studies have shown that PQ-induced cytotoxicity is associated with oxidative stress, including lipid peroxidation and ROS production. Oxidative stress is caused by a redox state characterized by dysfunction or an imbalance of excess free radicals in the antioxidant system (He et al., 2016; Huang et al., 2016). These highly toxic free radicals are extremely destructive to cells and human organs. Therefore, monitoring ROS production and ROS-induced changes in antioxidant systems is a common method of assessing oxidative stress (Lushchak, 2011). The findings in our present study are consistent with those of previous reports, further demonstrating that oxidative stress plays a crucial role in PQ-induced cytotoxicity. Mitochondria are a major source of intracellular ROS and an important target of ROS damage (Castello et al., 2007). Our study revealed that the impairment of mitochondrial function indicated by the loss of the mitochondrial potential and the decline in ATP production occurred as a result of cellular oxidative stress. Apoptosis, as one of the major outcomes of PQ-induced cytotoxicity, may be the major mechanism underlying PQ-induced cell death. An *in vivo* animal study also demonstrated that PQ-induced hepatotoxicity is characterized by impaired liver function and the induction of hepatic oxidative stress, resulting in histopathological changes. In exploring the antagonism of 8-FOB against PQ-induced hepatotoxicity, we found that this compound could markedly reverse PQ-induced hepatotoxicity-related biochemical and morphological changes *in vitro* and *in vivo*. 8-FOB could serve as a potent antioxidant for the treatment of PQ intoxication, as indicated by its ability to inhibit PQ-induced early ROS generation, reduce lipid peroxidation, preserve mitochondrial function, protect liver function, and hence mitigate PQ-induced hepatotoxicity. The existence of other mechanisms underlying the protective effects of 8-FOB in antagonizing PQ hepatotoxicity needs further investigation. The pharmacological value of 8-FOB for the treatment of other diseases also remains to be explored.

In summary, the present study demonstrated that 8-FOB is a potent antioxidant for protection against PQ-induced hepatotoxicity. These results shine new light on the understanding of the biological and pharmacological actions of this homoisoflavonoid compound. Moreover, the present study revealed the great potential of flavonoids as protective agents for treating PQ intoxication in clinical practice.

## Data Availability Statement

The datasets generated for this study are available on request to the corresponding author.

## Ethics Statement

Male C57BL/6 mice aged 8 weeks, weighing 20–24 g, were purchased from the animal centre of Army Medical University (Chongqing, China). Mice were routinely housed with free access to food and water. Generous efforts have been made to reduce the use and suffering of animals. All animal procedures and study protocols were approved by the Third Military Medical University Animal Care and Use Committee.

## Author Contributions

J-YQ initiated the project with guidance from ZZ and Z-PY. All experiments were performed by J-YQ, PD, LP, Y-DL, and L-LY. J-YQ, ZZ, and L-CW contributed to the analysis and interpretation of the data. J-YQ, ZZ, and PD prepared the figures and wrote the manuscript. Z-PY and ZZ conducted the critical revision of the manuscript and the approval of the article. All authors read and approved the final manuscript.

## Conflict of Interest

The authors declare that the research was conducted in the absence of any commercial or financial relationships that could be construed as a potential conflict of interest.
